# Modeling healthcare demands and long-term costs following pediatric traumatic brain injury

**DOI:** 10.3389/fneur.2024.1385100

**Published:** 2024-11-29

**Authors:** Jared G. Wiegand, Zorays Moazzam, Bruno P. Braga, Sarah E. Messiah, Faisal G. Qureshi

**Affiliations:** ^1^School of Public Health, University of Texas Health Science Center, Dallas, TX, United States; ^2^Department of Surgery, The Ohio State University Wexner Medical Center, Columbus, OH, United States; ^3^Children’s Health System of Texas, Dallas, TX, United States; ^4^Division of Pediatric Neurosurgery, Department of Neurosurgery, UT Southwestern Medical Center, Dallas, TX, United States; ^5^Center for Pediatric Population Health, UTHealth School of Public Health, Dallas, TX, United States; ^6^Department of Pediatrics, McGovern Medical School, Houston, TX, United States; ^7^Division of Pediatric Surgery, Department of Surgery, UT Southwestern Medical Center, Dallas, TX, United States

**Keywords:** traumatic brain injury, future treatment cost, future care, medical claims data, injury severity, pediatric

## Abstract

**Introduction:**

Traumatic brain injury (TBI) is a leading cause of death and disability in children, but data on the longitudinal healthcare and financial needs of pediatric patients is limited in scope and duration. We sought to describe and predict these metrics following acute inpatient treatment for TBI.

**Methods:**

Children surviving their initial inpatient treatment for TBI were identified from Optum’s deidentified Clinformatics® Data Mart Database (2007-2018). Treatment cost, healthcare utilization, and future inpatient readmission were stratified by follow-up intervals, type of claim, and injury severity. Both TBI-related and non-TBI related future cost and healthcare utilization were explored using linear mixed models. Acute inpatient healthcare utilization metrics were analyzed and used to predict future treatment cost and healthcare demands using linear regression models.

**Results:**

Among 7,400 patients, the majority suffered a mild TBI (50.2%). For patients with at least one-year follow-up (67.7%), patients accrued an average of 28.7 claims and $27,199 in costs, with 693 (13.8%) readmitted for TBI or non-TBI related causes. Severe TBI patients had a greater likelihood of readmission. Initial hospitalization length of stay and discharge disposition other than home were significant positive predictors of healthcare and financial utilization at one-and five-years follow-up. Linear mixed models demonstrated that pediatric TBI patients would accrue 21.1 claims and $25,203 in cost in the first year, and 9.4 claims and $4,147 in costs every additional year, with no significant differences based on initial injury severity.

**Discussion:**

Pediatric TBI patients require long-term healthcare and financial resources regardless of injury severity. Our cumulative findings provide essential information to clinicians, caretakers, researchers, advocates, and policymakers to better shape standards, expectations, and management of care following TBI.

## Introduction

Traumatic Brain Injury (TBI) is a leading cause of death and disability for children in the United States and worldwide (1–4). Annual emergency room visits for pediatric TBI surpass 400,000 in the United States, with 50,000–60,000 subsequent hospitalizations (5). Despite survival rates of greater than 95%, recovery is prolonged with long-term disability and health implications (1, 6). These factors complicate the understanding of future healthcare utilization and long-term costs following initial discharge (7–10).

Most studies are restricted to convenience samples from emergency rooms or hospital admissions, with a focus on clinical effects rather than healthcare utilization (5, 11–15). Consequently, there is limited long-term healthcare utilization data after the first year post-injury (5, 15–20). This information is essential for both clinical and public health professionals as standards for care and other policies are evaluated to manage pediatric TBI patients. Furthermore, a better understanding of such needs may help set expectations for family members and other caretakers (21, 22).

With increased access to medical claims data (MCD), investigators can longitudinally follow and quantify healthcare resource utilization, patient diagnoses, and any associated costs throughout an individual’s insurance plan membership (23). Expanded evaluation of MCD for children following TBI provides a broader understanding of resource demands and its predictors (24). To our knowledge, MCD has not been used to study long-term healthcare utilization and financial costs after pediatric TBI with prior literature limited to one year post-injury in population-level analyses and other convenience samples (25, 26).

Using a nationwide patient sample over a potential ten-year follow-up period, we sought to leverage MCD to measure and model the long-term healthcare utilization and treatment costs of pediatric TBI patients. Additionally, the impact of TBI severity on future consumption of healthcare resources was evaluated. We hypothesized that pediatric TBI patients will continue to utilize healthcare resources and accrue costs over time, and that increasing TBI severity will contribute to higher treatment costs.

## Materials and methods

### Study population

Optum’s de-identified Clinformatics® Data Mart Database (2007–2018) was used to identify medical claims of pediatric patients with TBI. This commercial medical claims database includes all diagnostic, treatment, and financial information through inpatient, outpatient and pharmaceutical claims for over 65-million individuals nationwide over 12 years (27). Children were included based on International Classification of Disease Control and Prevention (ICD-9 and ICD-10) codes for a pediatric TBI suffered before their 18th birthday (Supplementary Table 1) (28). Only children who survived acute inpatient care for an initial TBI and received any inpatient or outpatient future care were included. Children only receiving outpatient care, those with missing demographic or diagnostic information were excluded in line with prior literature (Supplementary Figure 1) (25, 29). Approval of this analysis was obtained through the University of Texas Health Science Center at Houston (UTHealth) Committee for the Protection of Human Subjects (# HSC-SPH-19-0415).

### Measures

Patient demographics including sex, age at inpatient admission, and geographic region (northeast, central, south, west) were tabulated. TBI severity (mild, moderate, severe) was extrapolated from diagnoses codes using ICDPC-R statistical package for R statistical software (30). This software uses an algorithm to generate Abbreviated Injury Scale (AIS) scores that can then be used to extrapolate severity. Specifically, for ICD-9 and ICD-10 codes, ICDPC-R generates an approximate AIS, body region (head and neck, face, chest, abdomen, and pelvic contents, extremities, and pelvic bones, and general), and injury severity score. If a patient had multiple TBI diagnoses within their initial inpatient treatment, severity was defined using the most severe diagnoses. This methodology was previously verified among TBI patients using a large trauma registry (31).

Initial inpatient healthcare measures extracted from MCD included length of stay (LOS) during the initial inpatient admission (days), discharge status (home, facility, other), and total cost of inpatient stay (US dollars). Other healthcare utilization metrics based on organ system and clinical procedures were defined using ICD procedural codes and analyzed as any use (yes) or no use (no) within a claim. These were classified as surgical, diagnostic, or other treatment based on clinical review and further stratified into organ system specific classes including: bone/musculoskeletal, GI/digestive, neurological, plastic, pulmonary, and skin.

Future care after discharge was aggregated as TBI-related, non-TBI related or total. TBI-related measurements were identified with a TBI-related ICD code (28). Future care measures included number of claims, cost of care (US dollars) and inpatient readmission (yes vs. no). All cost variables represent charges and were standardized to the US dollar value in the 2018 fiscal year. Readmission was measured within one-, five-, and ten-years of follow-up. Cost and visits were measured on yearly intervals as accrued within a child’s follow-up period. Each patient’s cumulative follow-up period was measured as the time between their initial acute inpatient TBI diagnosis and most-recent medical claim.

### Statistical analyses

Continuous variables were presented as mean and 95% confidence intervals (95% CI), while categorical variables were presented as frequencies and percentages. Comparisons by TBI severity groups between continuous and categorical variables were made using ANOVA and chi-square tests, respectively. Healthcare utilization was analyzed during the initial inpatient treatment and future care periods. Demographics, healthcare utilization, and other variables of interest were tabulated in aggregate and by TBI severity.

Acute inpatient healthcare utilization variables were used as predictors for future demands using bivariable and multivariable linear regression models. Variables were included in multivariable models if they were considered demographic or statistically significant during bivariable analyses. Beta regression coefficients (*β*) and their standard errors were tabulated for one-, five-, and ten-year intervals post initial TBI treatment to measure the effect of each independent variable (characteristics) on the dependent variable (future costs and claims).

Linear mixed-models (LMM) were used to model future annual claims and costs starting one year after discharge from initial admission. Annual intervals were chosen to best quantify long-term accrual of data. Future claims and costs were modeled as TBI-related, non-TBI related, and total, and stratified by TBI severity when appropriate. LMM is advantageous in modeling these metrics over time as it adjusts for any random variation that may exist between patients. This adjustment can serve as a proxy for geographic or extraneous influences on future claims and costs Intercept and yearly beta parameter estimates (*β*) with confidence intervals, random effects standard deviation (RE SD), and fixed effects standard deviation (FE SD) were analyzed. Intercept estimates represent the accrued costs or claims within the first year, whereas year estimates represent the accrued costs or claims within every subsequent year. LMM assumptions of linearity, absence of collinearity, homoscedasticity, normality of residuals, and independence were validated prior to construction. All tests were two-sided and used a significance level of *α* = 0.05. All statistical analyses were conducted using R 3.6.1 (32).

## Results

### Demographics and initial inpatient treatment

Seventy-four hundred patients were identified nationally with the greatest number of patients from the southern United States (Supplementary Table 2). The mean age was 9.6 years and the majority of patient were male (*n* = 4,879, 65.9%) and had suffered a mild TBI (3,720, 50.2%) (Table 1). Notably, severe TBI patients were younger (9.0 years, 95% CI 8.7–9.3) than those with mild (9.6 years, 95% CI, 9.4–9.8) and moderate TBI (9.9 years, 95% CI 9.7–10.1) (*p* < 0.001).

**Table 1:** Pediatric TBI patient demographics, care received during initial admission, and follow-up time.

TBI severity					
Characteristic	All (*N* = 7,400)	Mild (*n* = 3,720)	Moderate (*n* = 2,624)	Severe (*n* = 1,056)	*p*-value
Age^a^, mean (95% CI)	9.6 (9.5, 9.7)	9.6 (9.4, 9.8)	9.9 (9.7, 10.1)	9.0 (8.7, 9.3)	<0.001
Sex, n (%)					0.37
Male	4,879 (65.9)	2,424 (65.2)	1,751 (66.7)	704 (66.7)	
Female	2,521 (34.1)	1,296 (34.8)	873 (33.3)	352 (33.3)	
Initial Inpatient Admission					
Length of Stay^b^, mean (95% CI)	4.0 (3.8, 4.2)	3.0 (2.8, 3.3)	4.6 (4.3, 4.8)	6.0 (5.4, 6.7)	<0.001
Discharge Status, n (%)					<0.001
Home	6,562 (88.7)	3,426 (92.1)	2,265 (86.3)	871 (82.5)	
Facility^c^	238 (3.2)	70 (1.9)	121 (4.6)	47 (4.5)	
Other/Unknown	600 (8.1)	224 (6.0)	238 (9.1)	138 (13.1)	
Total Cost of Care^d^, mean (95% CI)	$22,196 ($20,218, $24,174)	$15,978 ($14,357, $17,599)	$27,659 ($22,803, $32,516)	$30,529 ($26,889, $34,170)	<0.001
Diagnostic Procedure, n (%)					
CT Scan	5,594 (75.6)	2,615 (70.3)	2,111 (80.4)	868 (82.2)	<0.001
MRI	1,263 (17.1)	613 (16.5)	390 (14.9)	260 (24.6)	<0.001
X-Ray	4,175 (56.4)	1,993 (53.6)	1,538 (58.6)	644 (61.0)	<0.001
Surgical Procedure, n (%)	1,669 (22.6)	681 (18.3)	678 (25.8)	310 (29.4)	<0.001
System Specific Procedure, n (%)					
Bone/Musculoskeletal	718 (9.7)	317 (8.5)	289 (11.0)	112 (10.6)	0.002
Vascular	607 (8.2)	168 (4.5)	280 (10.7)	159 (15.1)	<0.001
Neurological	581 (7.9)	194 (5.2)	221 (8.4)	166 (15.7)	<0.001
Skin	580 (7.8)	251 (6.7)	254 (9.7)	75 (7.1)	<0.001
Plastic	229 (3.1)	71 (1.9)	138 (5.3)	20 (1.9)	<0.001
Pulmonary	183 (2.5)	40 (1.1)	101 (3.8)	42 (4.0)	<0.001
Gastrointestinal/Digestive	166 (2.2)	50 (1.3)	79 (3.0)	37 (3.5)	<0.001
Follow-Up Post Admission					
Total^e^, mean (95% CI)	2.6 (2.6, 2.7)	2.6 (2.6, 2.7)	2.7 (2.6, 2.8)	2.5 (2.4, 2.6)	0.24
At Least 1 Year, n (%)	5,011 (67.7)	2,560 (68.8)	1,761 (67.1)	690 (65.3)	0.07
At Least 5 Years, n (%)	1,775 (24.0)	882 (23.7)	651 (24.8)	242 (22.9)	0.41
At Least 10 Years, n (%)	211 (2.9)	104 (2.8)	77 (2.9)	30 (2.8)	0.95

TBI, Traumatic Brain Injury; CT Scan, Computerized Tomography Scan; MRI, Magnetic Resonance Imaging.

a Age at initial TBI diagnosis is reported in years.

b Length of stay is reported in days.

c Facility includes: long-term care, psychiatric, rehab, and skilled nursing facilities.

d Costs were reported in dollars (standardized to the fiscal year 2018) and rounded to the nearest whole dollar.

e Total follow-up post admission is reported in years.

During initial inpatient treatment for TBI, the mean length of stay (LOS) was 4.0 days with highest LOS for severe TBI patients (6.0 days, 95% CI, 5.4–6.7). Most patients (*n* = 6,562, 88.7%) were discharged home; however, increasing severity was associated with lower rates of discharge to home (*p* < 0.001). The mean cost of a patient’s initial admission was $22,196, with highest costs for severe TBI patients ($30,529, 95% CI $26,889–$34,170; *p* < 0.001). During initial admission, severe TBI patients had the highest percentage of health care utilization for all diagnostic and surgical procedures (*n* = 310, 29.4%) and the highest percentage of neurological (*n* = 166, 15.7%), vascular (*n* = 159, 15.1%), pulmonary (*n* = 42, 4.0%), and gastrointestinal procedures (*n* = 37, 3.5%).

### Future health care utilization and cost

Patients had a mean follow-up of 2.6 years after discharge (Table 1). Of note, 5,011 (67.7%) patients had at least 1 year of follow-up, with 1,775 (24%) patients having at least 5 years of follow-up. Only 211 (2.9%) of patients had follow-up of 10 years or more. No statistical differences were noted in follow-up periods between TBI severity groups (*p* = 0.24).

At 1 year follow up, patients had 28.7 claims during that time, at a cost of $27,199 (Table 2). Notably, 693 (13.8%) patients required readmission within the first year, with roughly half undergoing a TBI-related readmission. At the one-year mark, patients with severe TBI had a significantly greater likelihood of being readmitted for TBI (*p* < 0.002). Claims, costs, and readmissions continued to rise for both TBI and non-TBI causes at 5- and 10-years of follow-up, with no significant impact of initial TBI severity (Supplementary Tables 3, 4).

**Table 2:** Future health care utilization, treatment cost, and inpatient readmission within one-, five-, and ten-year intervals for pediatric TBI patients.

	Follow-up interval		
Characteristic	1-year (*n* = 5,011)	5-years (*n* = 1,775)	10-years (*n* = 211)
Future Claims, mean (95% CI)			
Total	28.7 (27.9, 29.5)	67.8 (65.9, 69.8)	109.6 (105.9, 113.3)
TBI^a^	7.5 (7.2, 7.8)	10.7 (10.0, 11.5)	12.9 (12.0, 13.8)
Non-TBI^b^	22.8 (22.2, 23.5)	59.0 (57.4, 60.6)	96.2 (93.5, 98.8)
Future Cost^c^, mean (95% CI)			
Total	$27,199 ($25,238, $29,161)	$44,069 ($41,576, $46,561)	$51,056 ($48,840, $53,271)
TBI^a^	$13,614 ($12,542, $14,685)	$16,392 ($15,117, $17,668)	$15,732 ($14,499, $16,965)
Non-TBI^b^	$16,204 ($14,592, $17,817)	$30,564 ($28,563, $32,564)	$37,901 ($36,508, $39,293)
Future Inpatient Readmission, n (%)			
All	693 (13.8)	374 (21.1)	51 (24.2)
Mild	312 (12.2)	189 (21.4)	26 (25.0)
Moderate	261 (14.8)	136 (20.9)	16 (20.8)
Severe	120 (17.4)	49 (20.2)	9 (30.0)
Future TBI^a^ Inpatient Readmission, n (%)			
All	338 (6.7)	139 (7.8)	13 (6.2)
Mild	134 (5.2)^d^	61 (6.9)	4 (3.8)
Moderate	137 (7.8)^d^	54 (8.3)	6 (7.8)
Severe	67 (9.7)^d^	24 (9.9)	3 (10.0)
Future Non-TBI^b^ Inpatient Readmission, n (%)			
All	460 (9.2)	300 (11.7)	42 (19.9)
Mild	220 (8.6)	156 (17.7)	22 (21.1)
Moderate	165 (9.4)	107 (16.4)	13 (16.8)
Severe	75 (10.9)	37 (15.3)	7 (23.3)

TBI, Traumatic Brain Injury.

a TBI-related measurements were identified as claims with a TBI-related ICD code.

b Non-TBI related measurements were identified as claims without a TBI-related ICD code.

c Costs were reported in dollars (standardized to the fiscal year 2018) and rounded to the nearest whole dollar.

d Chi Square tests identified significant (*p* < 0.05) differences between TBI severity groups.

### Future predictions of health care utilization and cost

Results from bivariable linear regression models to predict future claims and treatment cost are tabulated in Supplementary Tables 5–10. TBI severity, age at TBI diagnosis, sex, geographic region, LOS, discharge status, and receipt of diagnostic and surgical procedures during admission treatment were used to adjust multivariable modes accordingly based on statistical significance (*p* < 0.05) (Table 3).

**Table 3:** Multivariable linear regression models to predict future claims and treatment costs within one-, five-, and ten-year intervals based on initial admission.

	Year post TBI								
	1-year (*n* = 5,011)			5-years (*n* = 1,775)			10-years (*n* = 211)		
Characteristic	β^e^	SE	*p*-value	β^e^	SE	*p*-value	β^e^	SE	*p*-value
Future Claims									
Intercept	29.4	1.7	<0.001	79.7	7.2	<0.001	71.4	30.4	0.02
Demographics									
Age^a^	0.2	0.01	0.06	0.1	0.4	0.81	−1.9	2.3	0.41
Sex									
Male	Reference	–	–	Reference	–	–	Reference	–	–
Female	2.5	1.2	0.03	0.66	4.8	0.90	55.9	25.8	0.03
TBI Severity									
Mild	Reference	–	–	Reference	–	–	Reference	–	–
Moderate	−5.2	1.2	<0.001	−12.45	5.1	0.02	−5.2	27.3	0.85
Severe	−2.5	1.7	0.13	−10.92	7.2	0.13	−57.2	38.7	0.14
Initial Inpatient Care									
Length of Stay^b^	7.5	0.1	<0.001	2.8	0.3	<0.001	7.2	1.4	<0.001
Discharge Status									
Home	Reference	–	–	Reference	–	–	x^f^	x^f^	x^f^
Facility^c^	45.9	3.3	<0.001	42.1	13.9	0.002	x^f^	x^f^	x^f^
Other/Unknown	12.6	2.3	<0.001	11.9	9.8	<0.001	x^f^	x^f^	x^f^
Surgical Procedure	3.7	1.5	0.01	6.1	6.5	0.35	x^f^	x^f^	x^f^
Future Costs^d^									
Intercept	$1,940	$440	<0.001	$36,170	$9,211	<0.001	$32,517	$20,968	0.12
Demographics									
Age^a^	$493	$232	0.03	$1,110	$508	0.003	$230	$15,597	0.86
Sex									
Male	Reference	–	–	Reference	–	–	Reference	–	–
Female	$1,059	$2,922	0.72	-$5,565	$6,164	0.37	$29,713	$15,597	0.06
TBI Severity									
Mild	Reference	–	–	Reference	–	–	Reference	–	–
Moderate	$343	$3,064	0.92	-$5,073	$6,523	0.44	-$13,946	$16,023	0.39
Severe	$258	$4,257	0.95	$8,630	$9,112	0.34	$24,048	$22,690	0.29
Initial Inpatient Care									
Length of Stay^b^	$1,270	$161	<0.001	$2,092	$423	<0.001	$4,286	$969	<0.001
Discharge Status									
Home	Reference	–	–	Reference	–	–	Reference	–	–
Facility^c^	$108,300	$8,256	<0.001	$111,700	$17,660	0.23	$183,212	$57,230	0.002
Other/Unknown	$29,610	$5,873	<0.001	$16,990	$12,500	0.17	-$5,745	$33,710	0.87
Surgical Procedure	$2,724	$3,766	0.47	$3,554	$8,314	0.67	$24,414	$23,397	0.30

TBI, Traumatic Brain Injury; SE, Standard Error.

a Age at initial TBI diagnosis is reported in years.

b Length of stay is reported in days.

c Facility includes: long-term care, psychiatric, rehab, and skilled nursing facilities.

d Costs were reported in dollars (standardized to the fiscal year 2018) and rounded to the nearest whole dollar.

e Beta represents the effect size or impact of each independent variable (characteristics) on the dependent variable (future costs or future claims).

f Variable was dropped from the multivariate linear regression model because it was not a demographic nor significant variable when ran as a bivariable linear regression model.

Notably, initial LOS during acute admission treatment was a significant positive predictor for future claims and treatment cost at all follow-up intervals following multivariable adjustment (*p* < 0.001). Discharge to a facility other than home also predicted significantly higher future claims at the one-year (45.9, SE, 3.3, *p* < 0.001) and five-years (42.1, SE, 13.9, *p* < 0.05) follow-up, but only significantly higher future costs at the one-year ($108,300, SE, $8,256, *p* < 0.001) and 10-years ($183,212, SE, $57,230, *p* = 0.002) intervals. Furthermore, receipt of surgical procedures at the initial inpatient admission predicted significantly higher claims within the first year (3.7, SE, 1.5, *p* = 0.01), but not costs after adjustment.

After adjustment, TBI severity did not increase claims or costs at long-term follow-up. Similarly, age was not a significant predictor for future claims at any follow-up interval after multivariable adjustment, but did increase costs at one-year ($493, SE, $232, *p* = 0.03) and five-years ($1,110, SE, $508, *p* = 0.003). When compared with male patients, females had significantly increased future claims within one-year (2.5, SE, 1.2, *p* = 0.03) and ten-years of follow up (55.9, SE, 25.8, *p* = 0.03). However, patient sex did not impact future cost at any follow-up interval.

### Linear mixed modeling of future medical claims and cost

Models of total claims over time showed that on average patients will accrue 21.1 (95% CI 19.0–23.1) total claims within one-year of follow-up with considerable patient-level variation (RE SD = 65.0), with 9.4 (95% CI 9.1–9.7) additional claims in each subsequent year (Table 4). Of these, 32% were related to TBI (6.9, 95% CI 6.0–7.8) within the first year of treatment. However, TBI-related claims represented 7.4% (0.7 (95% CI 0.6–0.9)) of total annual claims per year every additional year after. Total cost was modeled over time to show that on average, patients accrued $25,203 (95% CI $20,139–$30,267) in medical cost within their first year of treatment with considerable patient-level variation (RE SD = $169,337), with $4,147 (95% CI $3,574–$4,722) additional cost each subsequent year. Of these costs, 52% were related to TBI ($13,084, 95% CI $11,274–$14,895) within the first year of treatment. TBI-related cost increased by $566 (95% CI $478–$653) yearly, but only represented 13.6% of the patient’s cost of care as each additional year progressed.

**Table 4:** Linear mixed model parameters for future medical claims and treatment costs following pediatric TBI.

Model	Parameter name	β^d^	Lower 95% confidence interval	Upper 95% confidence interval	Random effects standard deviation	Fixed effects standard deviation
Future Claims						
Total	Intercept (Patient)	21.1	19.0	23.1	65.0	1.0
	Year	9.4	9.1	9.7	–	0.1
TBI^a^	Intercept (Patient)	6.9	6.0	7.8	22.2	0.4
	Year	0.7	0.6	0.9	–	0.1
Non-TBI^b^	Intercept (Patient)	15.4	13.7	17.1	52.3	0.9
	Year	8.8	8.6	9.1	–	0.1
Future Costs^c^						
Total	Intercept (Patient)	$25,203	$20,139	$30,267	$168,337	$2,583
	Year	$4,147	$3,574	$4,722	–	$293
TBI^a^	Intercept (Patient)	$13,084	$11,274	$14,895	$58,923	$924
	Year	$566	$478	$653	-	$45
Non-TBI^b^	Intercept (Patient)	$14,320	$9,575	$19,064	$155,792	$2,421
	Year	$3,691	$3,126	$4,257	–	$288

TBI, Traumatic Brain Injury.

a TBI-related measurements were identified as claims with a TBI-related ICD code.

b Non-TBI related measurements were identified as claims without a TBI-related ICD code.

c Costs were reported in dollars (standardized to the fiscal year 2018) and rounded to the nearest whole dollar.

d Beta represents the effect size or impact of each independent variable (characteristics) on the dependent variable (future costs or future claims).

**Figure 1:**
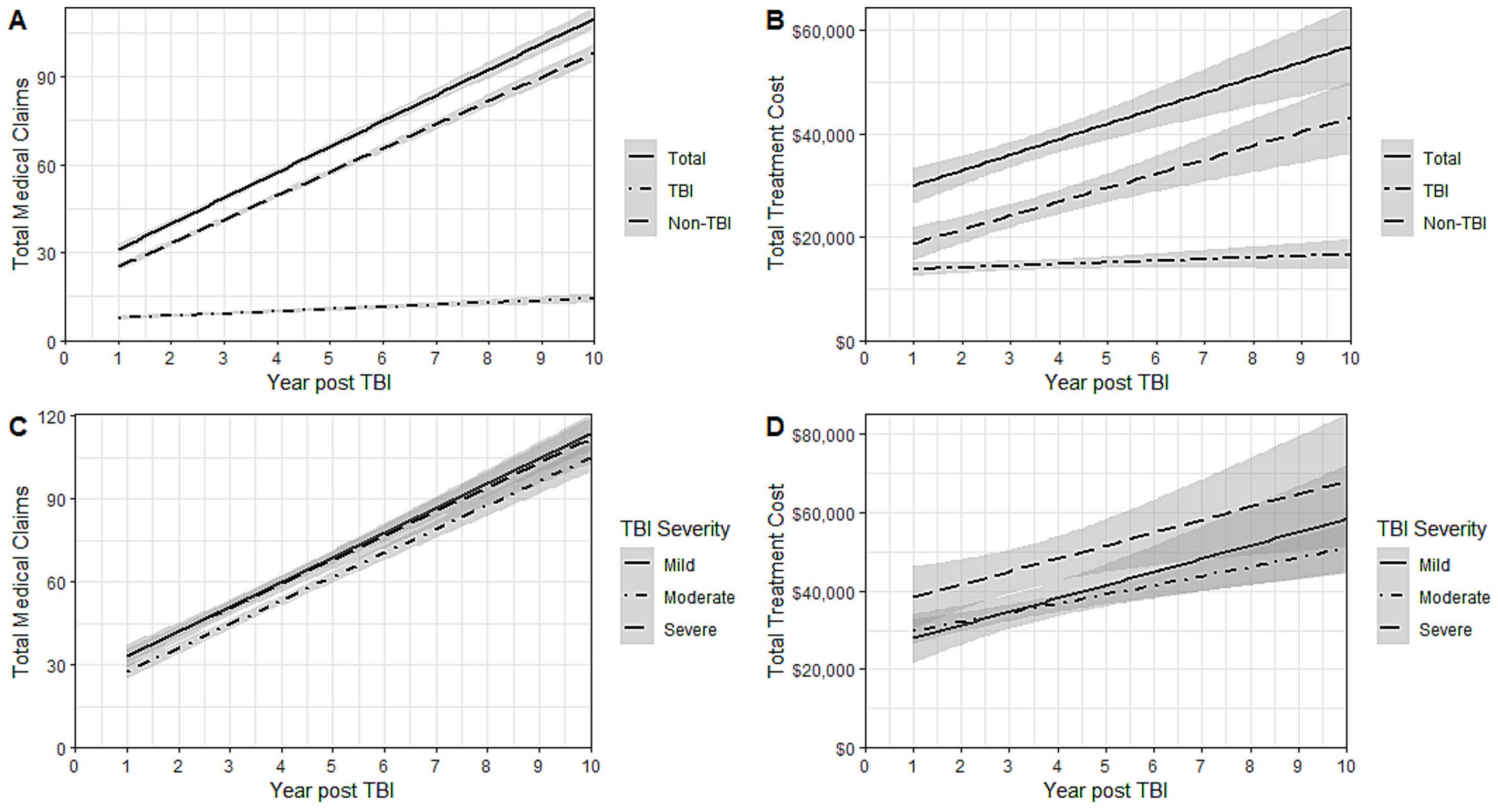
Linear mixed models for **(A)** total medical claims by TBI-relatedness **(B)** total treatment costs^a^ by TBI-relatedness **(C)** total medical claims by TBI Severity at initial diagnosis **(D)** total treatment costs^a^ TBI Severity at initial diagnosis. TBI, Traumatic Brain Injury. ^a^Costs were reported in dollars (standardized to the fiscal year 2018) and rounded to the nearest whole dollar.

LMM for total claims and costs by TBI-relatedness and severity are illustrated over time in (Figure 1). While no significant differences for total claims and costs by TBI persisted through 10-years of follow-up, LMM for total cost showed increased variability compared with claims due to TBI severity (Supplementary Table 11 and Supplementary Figure 2). These models demonstrated clear positive accrual of claims and costs over time for all patients, regardless of TBI severity at the time of initial admission.

## Discussion

In the present study, pediatric TBI patients demanded significant acute and long-term resources, regardless of injury severity over time. Specifically, our LMM analysis demonstrated that pediatric TBI patients would accrue 21.1 claims and $25,203 in financial cost in the first year, and 9.4 claims and $4,147 every subsequent year. Our results echo prior findings that reflect high initial inpatient expenditures followed by prolonged recovery after TBI (9). Additionally, our results highlight the difficulty of following this patient population over time, as roughly one-third of patients were lost to follow-up after 1 year post-injury. This may illustrate the difficulty pediatric patients face in receiving follow-up care. Initial treatment LOS and discharge other than home were significant positive predictors of healthcare and financial utilization at one- and five-years of follow-up, stressing the impact of acute care metrics on long-term utilization and costs.

Previous study cohorts and follow-up periods have been limited in their attempt to quantify healthcare utilization and associated costs after TBI (5, 8, 11, 14–19, 22). The lack of adequate follow-up after pediatric TBI may be due to an emphasis on subjective metrics of quality of life and function, rather than objective burden of disease measurements (33–35). Our analysis illustrates the utility of MCD as an objective measure and proxy for healthcare utilization, allowing for a more comprehensive view of the future care and cost demands of pediatric TBI patients.

Among our cohort, 50% were diagnosed with mild TBI, while 36 and 14% were diagnosed with moderate and severe TBI. This is congruent with prior findings that mild TBI accounts for 31.0–68.6% of admissions after pediatric TBI cases (14, 16, 22, 26). Moderate-to-severe TBIs were also more common in the southern U.S. among our cohort, which has been reported previously (Supplementary Table 2) (13, 36–40). It is important to note, however, that our selection criteria excluded patients who sought care during their initial diagnosis of a pediatric TBI in an outpatient environment. Consequently, our results cannot be extrapolated to pediatric TBI managed strictly in an outpatient setting (41).

As anticipated, TBI severity predicted utilization and costs during initial admission, including increased use of diagnostic tools and procedures, longer LOS, and higher likelihood of discharge outside the home. Furthermore, patients with severe TBI were more likely to experience a TBI-related readmission within 1 year after discharge. Prior studies report similar results, with increased severity as a strong risk factor for revisits and readmissions (10). However, TBI severity had no significant impact on long-term healthcare utilization or costs, with both accruing over time for all patients, regardless of severity of injury. Our results may illustrate an increase in the cost of claims due to increased TBI severity, as TBI utilization claims were lower than treatment costs for these patients.

Notably, a longer initial LOS and discharge to a facility rather than home after initial admission were positively associated with claims and costs at one-, five-, and 10-years follow-up, as reflected through the inclusion of patients who suffered a moderate or severe TBI. Although these associations were previously documented, the duration of patient follow-up was generally limited to one-year post-discharge (20, 33, 42). The present study’s longer duration of patient follow-up of potentially ten-years is a key distinguishing factor from prior studies with these findings, as data is generally limited following initial hospitalization.

Although roughly two-thirds of patients were followed for at least one-year, this number rapidly decreased thereafter, with a quarter followed for at least 5-years and only 2.9% remaining after 10-years. This phenomenon is likely multi-factorial and subject to changes, such as a switch in a patient’s or family’s insurance status over the study period.

To minimize the impact of an incrementally decreased sample size on the ability to model long-term outcomes, we utilized linear mixed modeling (LMM). A key advantage of this statistical technique is that it takes into account inter-individual variability (random effects) (43–45). Our models illustrate that total claims continue to increase linearly with time, with the majority of claims being non-TBI during subsequent follow-up. Although TBI-related claims accounted for a third of total claims within the first year, only 7.4% of all new claims each subsequent year were TBI-related. Similarly, TBI-related costs accounted for 51.9% of total costs within the first year, followed by only 13.6% of all new costs each additional year. This echoes prior findings that nearly 53% of total costs incurred post-TBI were concentrated in the first year post-admission (10). Furthermore, it is important to note that increased claims did not directly translate to higher costs.

Our results also highlight the substantial financial and resource demands within the first year after pediatric TBI. After one year of follow-up, healthcare costs amounting to $4,147 were spent annually, although only $565 were TBI-related costs. This finding was particularly interesting, as we did not expect non-TBI costs to markedly outpace TBI-related costs. However, this may be explained by the rapid increase in US healthcare costs across all sectors over the preceding decade, with healthcare spending on children increasing from $149.6 billion in 1996 to $233.5 billion by 2013 or patients not receiving the proper education or follow-up following their initial TBI hospitalization (46, 47).

This study must be interpreted with an understanding of its limitations. Firstly, patients were identified through ICD codes in an administrative database and classified using ICDPC-R statistical package; thus, misclassification bias was possible. Specifically, the lack of association between TBI severity and utilization/costs may signify a limitation in the ICDPC-R algorithm. Furthermore, although Optum’s de-identified Clinformatics® Data Mart Database represents over 65 million patients, these findings may not be generalizable to all pediatric patients admitted for TBI with other modes of insurance and puts the study at risk of information bias. Although exclusion of pediatric TBI patients who were not admitted for inpatient treatment resulted in large number omitted from the analysis, this was critical to homogenize our cohort to those only treated initially in an inpatient setting. Future studies may consider assessing pediatric TBI patients regardless of inpatient stay such as those discharged from the emergency department for a more holistic analysis of pediatric TBI patients. Additionally, we used non-TBI related procedures as a surrogate for non-TBI injuries, rather than the actual injuries, as this would require very granular information on the specific injuries. This granularity was not available in the dataset and is a limitation. And although it appears that most long-term costs and claims are non-TBI related, it is important to note that additional claims may have occurred that possibly were not linked via coding to the original TBI diagnosis, and as such were analyzed as “non-TBI.” This may introduce potential information bias related to medical coding. Any costs incurred after initial TBI only included healthcare costs processed through an insurance provider and did not include indirect costs such as time missed from school or transportation, possibly underrepresenting the “true” long-term cost of TBI. Future studies should explore healthcare utilization and costs based on direct causal relation to the initial injury, aim to incorporate socioeconomic data and evaluate the impact of social determinants of health on healthcare utilization and costs in this patient population, and validate our results utilizing a matched control group. Finally, it is possible that as children transition to adulthood, their insurance status and insurance plans changed, leading them to drop out of this cohort and underestimate healthcare utilization and cost for all-severity TBI due to attrition bias. We also did not have access to post-discharge mortality data to adjust for death. These are issues inherently linked to MCD and thus could not be accounted for. However, we utilized MCD as an indicator of healthcare utilization and costs, allowing for the analysis of data over a potential 10-years of follow-up.

Ultimately, this study illustrates the patient-level burden of pediatric TBI in the United States as well as the long-term TBI and non-TBI financial and healthcare demands. Initial LOS and discharge status were the greatest positive predictors of healthcare utilization and costs over time. These longitudinal relationships can help patient stakeholders justify additional resources toward those with more demanding initial inpatient treatment or non-home discharge and highlight the need for a comprehensive strategy, including injury prevention, initial inpatient care, and long-term follow-up irrespective of initial TBI severity. Our findings also provide a quantifiable understanding of the unique long-term needs of the pediatric TBI patient population. With this information, clinicians and caretakers will have a greater perspective on the expectations and management of the healthcare needs of these patients. And researchers, advocates, and policymakers will be informed to shape public health policy as standards of care and preventive strategies evolve.

## Data Availability

The data analyzed in this study is subject to the following licenses/restrictions: Optum’s de-identified Clinformatics^®^ Data Mart Database (2007–2018). Requests to access these datasets should be directed to UTHealth Houston School of Public Health, Center for Health Care Data, https://uthealthchcd.quickbase.com/db/bthuk9tt6/form?a=nwr&originalQid=td&page=1&ifv=1.

## References

[1] CDC. Traumatic brain injury and concussion. Available at: https://www.cdc.gov/traumaticbraininjury/index.html.

[2] Curtin SC Tejada-Vera B Bastian BA. Deaths: leading causes for 2020. Natl Vital Stat Rep. (2023) 72:1–115. doi: 10.15620/cdc:133059 PMID: 38085308

[3] CDC. Surveillance report of traumatic brain injury-related hospitalizations and deaths by age group, sex, and mechanism of injury—United States, 2016 and 2017. (2021). Available at: https://stacks.cdc.gov/view/cdc/111900/cdc_111900_DS1.pdf.

[4] Gardner RC Yaffe K. Epidemiology of mild traumatic brain injury and neurodegenerative disease. Mol Cell Neurosci. (2015) 66:75–80. doi: 10.1016/j.mcn.2015.03.001, PMID: 25748121 PMC4461453

[5] Schneier AJ Shields BJ Hostetler SG Xiang H Smith GA. Incidence of pediatric traumatic brain injury and associated hospital resource utilization in the United States. Pediatrics. (2006) 118:483–92. doi: 10.1542/peds.2005-2588 16882799

[6] Babikian T Asarnow R. Neurocognitive outcomes and recovery after pediatric TBI: meta-analytic review of the literature. Neuropsychology. (2009) 23:283–96. doi: 10.1037/a0015268, PMID: 19413443 PMC4064005

[7] Corrigan JD Hammond FM. Traumatic brain injury as a chronic health condition. Arch Phys Med Rehabil. (2013) 94:1199–201. doi: 10.1016/j.apmr.2013.01.023 23402722

[8] Slomine BS ML MC Ding R EJ MK Jaffe KM Aitken ME. Health care utilization and needs after pediatric traumatic brain injury. Pediatrics. (2006) 117:e663–74. doi: 10.1542/peds.2005-1892, PMID: 16533894

[9] Jaffe KM Massagli TL Martin KM Rivara JB Fay GC Polissar NL. Pediatric traumatic brain injury: acute and rehabilitation costs. Arch Phys Med Rehabil. (1993) 74:681–6. doi: 10.1016/0003-9993(93)90024-5, PMID: 8328886

[10] Hsia RY Mannix RC Guo J Kornblith AE Lin F Sokolove PE. Revisits, readmissions, and outcomes for pediatric traumatic brain injury in California, 2005-2014. PLoS One. (2020) 15:e0227981. doi: 10.1371/journal.pone.0227981, PMID: 31978188 PMC6980591

[11] McCrea MA Giacino JT Barber J Temkin NR Nelson LD Levin HS. Functional outcomes over the first year after moderate to severe traumatic brain injury in the prospective, longitudinal TRACK-TBI study. JAMA Neurol. (2021) 78:982–92. doi: 10.1001/jamaneurol.2021.2043, PMID: 34228047 PMC8261688

[12] Dikmen SS Machamer JE Powell JM Temkin NR. Outcome 3 to 5 years after moderate to severe traumatic brain injury. Arch Phys Med Rehabil. (2003) 84:1449–57. doi: 10.1016/s0003-9993(03)00287-9 14586911

[13] Hu J Ugiliweneza B Meyer K Lad SP Boakye M. Trend and geographic analysis for traumatic brain injury mortality and cost based on market scan database. J Neurotrauma. (2013) 30:1755–61. doi: 10.1089/neu.2013.2857, PMID: 23642155

[14] Leibson CL Brown AW Hall Long K Ransom JE Mandrekar J Osler TM. Medical care costs associated with traumatic brain injury over the full spectrum of disease: a controlled population-based study. J Neurotrauma. (2012) 29:2038–49. doi: 10.1089/neu.2010.1713, PMID: 22414023 PMC3408240

[15] Levant S Chari K DeFrances C. National hospital care survey demonstration projects: traumatic brain injury. Natl Health Stat Report. (2016) 97:1–16. 27483022

[16] Reuter-Rice K Doser K Eads JK Berndt S. Pediatric traumatic brain injury: families and healthcare team interaction trajectories during acute hospitalization. J Pediatr Nurs. (2017) 34:84–9. doi: 10.1016/j.pedn.2016.12.017, PMID: 28081932 PMC5444971

[17] Humphreys I Wood RL Phillips CJ Macey S. The costs of traumatic brain injury: a literature review. Clinicoecon Outcomes Res. (2013) 5:281–7. doi: 10.2147/CEOR.S44625, PMID: 23836998 PMC3699059

[18] Rockhill CM Fann JR Fan MY Hollingworth W Katon WJ. Healthcare costs associated with mild traumatic brain injury and psychological distress in children and adolescents. Brain Inj. (2010) 24:1051–60. doi: 10.3109/02699052.2010.494586, PMID: 20597633

[19] Adelson PD Pineda J Bell MJ Abend NS Berger RP Giza CC. Common data elements for pediatric traumatic brain injury: recommendations from the working group on demographics and clinical assessment. J Neurotrauma. (2012) 29:639–53. doi: 10.1089/neu.2011.1952 21939389 PMC3289844

[20] Shi J Xiang H Wheeler K Smith GA Stallones L Groner J. Costs, mortality likelihood and outcomes of hospitalized US children with traumatic brain injuries. Brain Inj. (2009) 23:602–11. doi: 10.1080/02699050903014907, PMID: 19557562 PMC3819720

[21] Aitken ME ML MC Slomine BS Ding R Durbin DR Jaffe KM. Family burden after traumatic brain injury in children. Pediatrics. (2009) 123:199–206. doi: 10.1542/peds.2008-0607 19117883

[22] Nelson RE Ma J Cheng Y Ewing-Cobbs L Clark A Keenan H. Healthcare utilization and missed workdays for parents of children with traumatic brain injury. J Head Trauma Rehabil. (2019) 34:257–67. doi: 10.1097/HTR.0000000000000458, PMID: 30608307 PMC7147293

[23] Wilson AB. The benefit of using both claims data and electronic medical record data in health care analysis. (2012). Available at: https://www.optum.com/content/dam/optum/resources/whitePapers/Benefits-of-using-both-claims-and-EMR-data-in-HC-analysis-WhitePaper-ACS.pdf.

[24] Jiang JY Gao GY Li WP Yu MK Zhu C. Early indicators of prognosis in 846 cases of severe traumatic brain injury. J Neurotrauma. (2002) 19:869–74. doi: 10.1089/08977150260190456, PMID: 12184856

[25] Graves JM Rivara FP Vavilala MS. Health care costs 1 year after pediatric traumatic brain injury. Am J Public Health. (2015) 105:e35–41. doi: 10.2105/AJPH.2015.302744, PMID: 26270293 PMC4566536

[26] Maddux AB Sevick C Cox-Martin M Bennett TD. Novel claims-based outcome phenotypes in survivors of pediatric traumatic brain injury. J Head Trauma Rehabil. (2021) 36:242–52. doi: 10.1097/HTR.0000000000000646, PMID: 33656469 PMC8249306

[27] Optum. Optum’s de-identifed Clinformatics® Data Mart Database. (2007–2019). Available at: https://www.optum.com/en/business/life-sciences/real-world-data/ehr-data.html.

[28] Schootman M Buchman TG Lewis LM. National estimates of hospitalization charges for the acute care of traumatic brain injuries. Brain Inj. (2003) 17:983–90. doi: 10.1080/0269905031000110427 14514449

[29] Taylor CA Bell JM Breiding MJ Xu L. Traumatic brain injury-related emergency department visits, hospitalizations, and deaths - United States, 2007 and 2013. MMWR Surveill Summ. (2017) 66:1–16. doi: 10.15585/mmwr.ss6609a1, PMID: 28301451 PMC5829835

[30] Clark DE Black AW Skavdahl DH Hallagan LD. Open-access programs for injury categorization using ICD-9 or ICD-10. Inj Epidemiol. (2018) 5:11. doi: 10.1186/s40621-018-0149-8, PMID: 29629480 PMC5890002

[31] Greene NH Kernic MA Vavilala MS Rivara FP. Validation of ICDPIC software injury severity scores using a large regional trauma registry. Inj Prev. (2015) 21:325–30. doi: 10.1136/injuryprev-2014-041524, PMID: 25985974

[32] R Development Core Team. R: A language and environment for statistical computing. Vienna, Austria: R Foundation for Statistical Computing (2010).

[33] Maddux AB Pinto N Fink EL Hartman ME Nett S Biagas K. Postdischarge outcome domains in pediatric critical care and the instruments used to evaluate them: a scoping review. Crit Care Med. (2020) 48:e1313–21. doi: 10.1097/CCM.0000000000004595 33009099 PMC7708523

[34] Aspesberro F Fesinmeyer MD Zhou C Zimmerman JJ Mangione-Smith R. Construct validity and responsiveness of the pediatric quality of life inventory 4.0 generic Core scales and infant scales in the PICU. Pediatr Crit Care Med. (2016) 17:e272–9. doi: 10.1097/PCC.0000000000000727, PMID: 27261668

[35] Pinto NP Rhinesmith EW Kim TY Ladner PH Pollack MM. Long-term function after pediatric critical illness: results from the survivor outcomes study. Pediatr Crit Care Med. (2017) 18:e122–30. doi: 10.1097/PCC.0000000000001070, PMID: 28107265

[36] Graves JM Mackelprang JL Moore M Abshire DA Rivara FP Jimenez N. Rural-urban disparities in health care costs and health service utilization following pediatric mild traumatic brain injury. Health Serv Res. (2019) 54:337–45. doi: 10.1111/1475-6773.13096, PMID: 30507042 PMC6407359

[37] Stewart TC Gilliland J Fraser DD. An epidemiologic profile of pediatric concussions: identifying urban and rural differences. J Trauma Acute Care Surg. (2014) 76:736–42. doi: 10.1097/TA.0b013e3182aafdf5 24553542

[38] Kim K Ozegovic D Voaklander DC. Differences in incidence of injury between rural and urban children in Canada and the USA: a systematic review. Inj Prev. (2012) 18:264–71. doi: 10.1136/injuryprev-2011-040306, PMID: 22634742

[39] Wolf LL Chowdhury R Tweed J Vinson L Losina E Haider AH. State-level geographic variation in prompt access to care for children after motor vehicle crashes. J Surg Res. (2017) 217:75–83.e1. doi: 10.1016/j.jss.2017.04.034 28558908 PMC5603370

[40] Yue JK Upadhyayula PS Avalos LN Cage TA. Pediatric traumatic brain injury in the United States: rural-urban disparities and considerations. Brain Sci. (2020) 10:135. doi: 10.3390/brainsci10030135, PMID: 32121176 PMC7139684

[41] McKinlay A Grace RC Horwood LJ Fergusson DM Ridder EM MacFarlane MR. Prevalence of traumatic brain injury among children, adolescents and young adults: prospective evidence from a birth cohort. Brain Inj. (2008) 22:175–81. doi: 10.1080/02699050801888824, PMID: 18240046

[42] Moore M Jimenez N Graves JM Rue T Fann JR Rivara FP. Racial disparities in outpatient mental health service use among children hospitalized for traumatic brain injury. J Head Trauma Rehabil. (2018) 33:177–84. doi: 10.1097/HTR.0000000000000348, PMID: 29194176 PMC6110532

[43] Laird NM Ware JH. Random-effects models for longitudinal data. Biometrics. (1982) 38:963–74. doi: 10.2307/2529876 7168798

[44] Singer JD Willett JB. Applied longitudinal data analysis: modeling change and event occurrence. New York, NY: Oxford University Press (2003).

[45] Schober P Vetter TR. Descriptive statistics in medical research. Anesth Analg. (2019) 129:1445. doi: 10.1213/ane.0000000000004480 31743158

[46] Bui AL Dieleman JL Hamavid H Birger M Chapin A Duber HC. Spending on Children’s personal health care in the United States, 1996-2013. JAMA Pediatr. (2017) 171:181–9. doi: 10.1001/jamapediatrics.2016.4086, PMID: 28027344 PMC5546095

[47] Oyesanya TO Loflin C You H Kandel M Johnson K Strauman T. Design, methods, and baseline characteristics of the brain injury education, training, and therapy to enhance recovery (BETTER) feasibility study: a transitional care intervention for younger adult patients with traumatic brain injury and caregivers. Curr Med Res Opin. (2022) 38:697–710. doi: 10.1080/03007995.2022.2043657, PMID: 35174756 PMC9131748

