# Supplementary material for: Modeling healthcare demands and long-term costs following pediatric traumatic brain injury
Source: Front Neurol. 2024 Nov 29;15:1385100. doi: 10.3389/fneur.2024.1385100 (PMC11638116; doi:10.3389/fneur.2024.1385100)
Supplement: Supplementary file 1 [file Data_Sheet_1.docx]

**Title:** Modeling Healthcare Demands and Long-Term Costs for Pediatric Traumatic Brain Injury

**Supplementary Table 1.** International Classification of Disease Control and Prevention (ICD-9 and ICD-10) codes for a pediatric TBI.

**Supplementary Figure 1.** Sample selection flowchart for pediatric TBI patients within Optum’s Clinformatics® de-identified Data Mart Database (2007-2018).

**Supplementary Table 2.** Pediatric TBI patient geographic region.

**Supplementary Table 3.** Future health care utilization within one-, five-, and ten-year intervals for pediatric TBI patients by TBI severity.

**Supplementary Table 4.** Future treatment cost within one-, five-, and ten-year intervals for pediatric TBI patients by TBI severity.

**Supplementary Table 5.** Simple linear regression models to predict total future claims within one year based on initial inpatient treatment.

**Supplementary Table 6.** Simple linear regression models to predict total future claims within five years based on initial inpatient treatment.

**Supplementary Table 7.** Simple linear regression models to predict total future claims within ten years based on initial inpatient treatment.

**Supplementary Table 8.** Simple linear regression models to predict total future cost within one year based on initial inpatient treatment.

**Supplementary Table 9.** Simple linear regression models to predict total future cost within five years based on initial inpatient treatment.

**Supplementary Table 10.** Simple linear regression models to predict total future cost within ten years based on initial inpatient treatment.

**Supplementary Table 11**. Linear mixed model parameters for future medical claims and treatment costs following pediatric TBI by TBI diagnosis at initial admission.

**Supplementary Figure 2**. Linear mixed models of total future medical claims and future treatment cost.

**Supplementary Table 1.** International Classification of Disease Control and Prevention (ICD-9 and ICD-10) codes for a pediatric TBI.

| **ICD Code** | | | |
| --- | --- | --- | --- |
| \| 34882 \| \| --- \| \| 85140 \| \| 85141 \| \| 85142 \| \| 85143 \| \| 85144 \| \| 85145 \| \| 85146 \| \| 85150 \| \| 85151 \| \| 85152 \| \| 85153 \| \| 85154 \| \| 85155 \| \| 85156 \| | \| 85159 \| \| --- \| \| 85160 \| \| 85164 \| \| 85166 \| \| 85169 \| \| 85170 \| \| 85171 \| \| 85172 \| \| 85173 \| \| 85176 \| \| 85179 \| \| 801x^a^ \| \| 803x^a^ \| \| 804x^a^ \| \| 8514x^a^ \| | \| 8515x^a^ \| \| --- \| \| 8516x^a^ \| \| 8517x^a^ \| \| G9382 \| \| S00 \| \| S02.0 \| \| S02.1 \| \| S02.8 \| \| S061 \| \| S061X7^a^ \| \| S06287 \| \| S062X^a^ \| \| S062X0^a^ \| \| S062X4^a^ \| \|  \| | \| S063 \| \| --- \| \| S06317 \| \| S06327 \| \| S06337 \| \| S06347 \| \| S06357 \| \| S06367 \| \| S06377 \| \| S06387 \| \| S064X7^a^ \| \| S0667 \| \| S0697 \| \| S09 \| \| V1552 \| |
| 1. Any code with an X present represents all codes with the beginning number sequence. | | | |

**Supplementary Figure 1.** Sample selection flowchart for pediatric TBI patients within Optum’s de-identified Clinformatics® Data Mart Database (2007-2018).

**
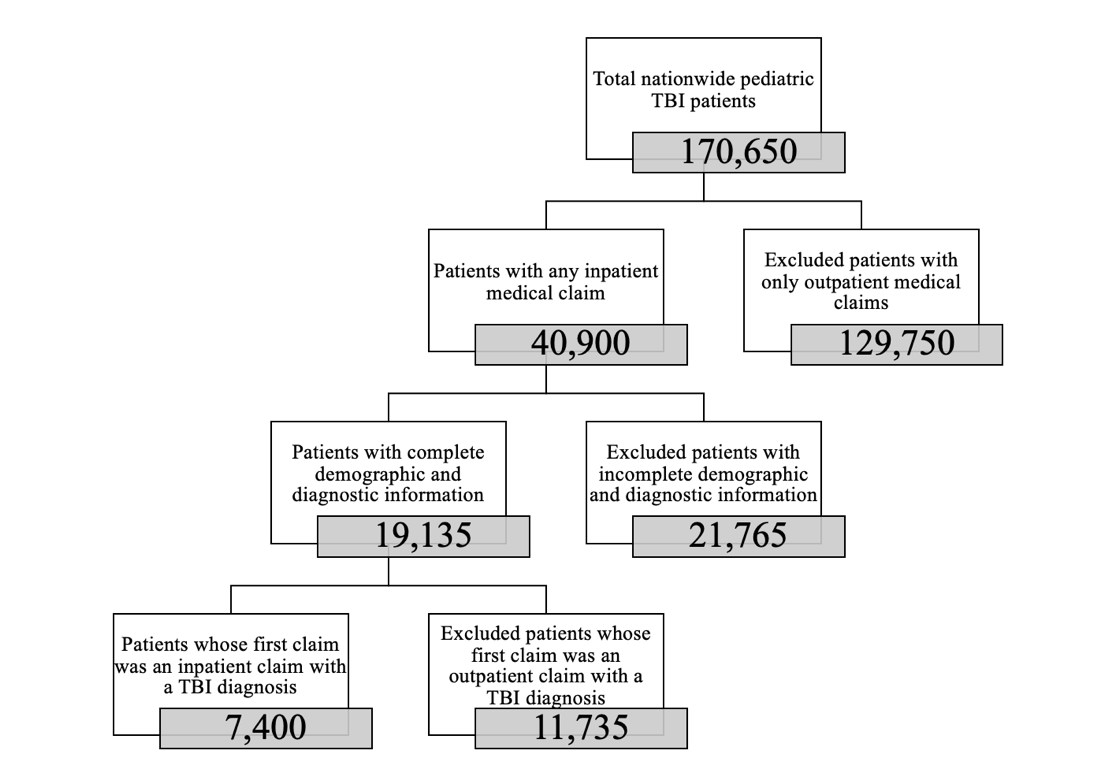
**

**Supplementary Table 2.** Pediatric TBI patient geographic region.

|  |  | **TBI Severity** | | |  |
| --- | --- | --- | --- | --- | --- |
| **Characteristic** | **All**  (N = 7,400) | **Mild**  (n = 3,720) | **Moderate**  (n = 2,624) | **Severe**  (n = 1,056) | **P-value**^a^ |
| Geographic Region, n (%) |  |  |  |  | < 0.001 |
| Northeast | 759 (10.2%) | 452 (59.5%) | 226 (29.8%) | 81 (10.7%) |  |
| Central | 1,832 (24.8%) | 902 (49.2%) | 654 (35.7%) | 276 (15.1%) |  |
| South | 3,006 (40.6%) | 1,447 (48.1%) | 1,098 (36.5%) | 461 (15.3%) |  |
| West | 1,803 (24.4%) | 919 (51.0%) | 646 (35.8%) | 238 (13.2%) |  |
| Abbreviation: TBI, Traumatic Brain Injury   1. Chi Square statistical test was used to assess differences between TBI severity groups. | | | | | |

**Supplementary Table 3.** Future health care utilization within one-, five-, and ten-year intervals for pediatric TBI patients by TBI severity.

|  | **Follow-Up Interval** | | |
| --- | --- | --- | --- |
| **Characteristic** | **1-year**  (n = 5,011) | **5-years**  (n = 1,775) | **10-years**  (n = 211) |
| **Future Claims** |  |  |  |
| Total, mean (95% CI) |  |  |  |
| All | 28.7 (27.9, 29.5) | 67.8 (65.9, 69.8) | 109.6 (105.9, 113.3) |
| Mild | 29.6 (28.3, 30.9)^c^ | 70.6 (67.6, 73.7) | 104.1 (98.7, 109.4) |
| Moderate | 26.2 (25.1, 27.3)^c^ | 63.7 (60.9, 66.4) | 120.4 (113.7, 127.1) |
| Severe | 31.7 (29.6, 33.8)^c^ | 68.6 (63.6, 73.7) | 100.9 (94.0, 107.9) |
| TBI^a^, mean (95% CI) |  |  |  |
| All | 7.5 (7.2, 7.8) | 10.7 (10.0, 11.5) | 12.9 (12.0, 13.8) |
| Mild | 6.8 (6.2, 7.3)^c^ | 10.1 (9.5, 10.6) | 15.7 (14.3, 17.1) |
| Moderate | 7.8 (7.3, 8.3)^c^ | 11.0 (10.6, 11.5) | 12.3 (10.8, 13.9) |
| Severe | 9.0 (8.2, 9.9)^c^ | 12.1 (11.2, 12.9) | 4.5 (4.3, 4.7) |
| Non-TBI^b^, mean (95% CI) |  |  |  |
| All | 22.8 (22.2, 23.5) | 59.0 (57.4, 60.6) | 96.2 (93.5, 98.8) |
| Mild | 24.7 (23.7, 25.7)^c^ | 63.0 (60.5, 65.6) | 90.5 (87.6, 93.4) |
| Moderate | 19.7 (18.8, 20.6)^c^ | 54.0 (51.7, 56.3) | 108.2 (102.4, 114.1) |
| Severe | 24.0 (22.4, 25.6)^c^ | 58.0 (54.0, 61.9) | 84.6 (79.9, 89.4) |
| Abbreviation: TBI, Traumatic Brain Injury   1. TBI-related measurements were identified as claims with a TBI-related ICD code. 2. Non-TBI related measurements were identified as claims without a TBI-related ICD code. 3. ANOVA statistical test assessed differences between TBI severity groups as statistically significant (p < 0.05). | | | |

**Supplementary Table 4.** Future treatment cost within one-, five-, and ten-year intervals for pediatric TBI patients by TBI severity.

|  | **Follow-Up Interval** | | |
| --- | --- | --- | --- |
| **Characteristic** | **1-year**  (n = 5,011) | **5-years**  (n = 1,775) | **10-years**  (n = 211) |
| **Future Cost**^a^ |  |  |  |
| Total, mean (95% CI) |  |  |  |
| All | $27,199 ($25,238, $29,161) | $44,069 ($41,576, $46,561) | $51,056 ($48,840, $53,271) |
| Mild | $23,883 ($20,836, $26,930)^d^ | $40,150 ($38,290, $43,009) | $47,416 ($44,757, $50,075) |
| Moderate | $29,205 ($26,125, $32,285)^d^ | $43,647 ($40,528, $46,766) | $53,171 ($49,145, $57,197) |
| Severe | $34,287 ($30,516, $38,058)^d^ | $59,503 ($47,278, $71,727) | $58,122 ($50,749, $65,495) |
| TBI^b^, mean (95% CI) |  |  |  |
| All | $13,614 ($12,542, $14,685) | $16,392 ($15,117, $17,668) | $15,732 ($14,499, $16,965) |
| Mild | $10,560 ($9,232, $11,888)^d^ | $13,468 ($11,654, $15,283) | $11,501 ($9,980, $13,021) |
| Moderate | $15,678 ($13,563, $17,792)^d^ | $18,609 ($16,410, $20,808) | $15,681 ($14,044, $17,318) |
| Severe | $18,200 ($15,746, $20,654)^d^ | $19,660 ($16,644, $22,677) | $30,867 ($25,229, $36,504) |
| Non-TBI^c^, mean (95% CI) |  |  |  |
| All | $16,204 ($14,592, $17,817) | $30,564 ($28,563, $32,564) | $37,901 ($36,508, $39,293) |
| Mild | $15,928 ($13,185, $18,670) | $29,856 ($27,884, $31,828) | $38,296 ($36,452, $40,141) |
| Moderate | $15,737 ($13,607, $17,867) | $27,227 ($25,334, $29,120) | $39,119 ($36,372, $41,866) |
| Severe | $18,426 ($16,083, $20,768) | $42,127 ($30,638, $53,615) | $33,429 ($30,878, $35,979) |
| Abbreviation: TBI, Traumatic Brain Injury   1. Costs were reported in dollars (standardized to the fiscal year 2018) and rounded to the nearest whole dollar. 2. TBI-related measurements were identified as claims with a TBI-related ICD code. 3. Non-TBI related measurements were identified as claims without a TBI-related ICD code. 4. ANOVA statistical test assessed differences between TBI severity groups as statistically significant (p < 0.05). | | | |

**Supplementary Table 5.** Simple linear regression models to predict total future claims within one year based on initial inpatient treatment.

|  | **1-year**  (n = 5,011) | | | |
| --- | --- | --- | --- | --- |
| **Model** | **Intercept** | **β** | **SE** | **p-value** |
| **Demographics** |  |  |  |  |
| Age^a^ | 23.6 | 0.5 | 0.1 | < 0.001 |
| Sex | 27.6 |  |  |  |
| Male |  | Reference | - | - |
| Female |  | 3.3 | 1.3 | 0.01 |
| Geographic Region | 29.3 |  |  |  |
| Northeast |  | -0.9 | 2.1 | 0.67 |
| Central |  | 2.9 | 1.9 | 0.06 |
| South |  | Reference | - | - |
| West |  | -5.3 | 1.6 | < 0.001 |
| TBI Severity | 29.6 |  |  |  |
| Mild |  | Reference | - | - |
| Moderate |  | -3.4 | 1.3 | 0.009 |
| Severe |  | 2.1 | 1.8 | 0.26 |
|  |  |  |  |  |
| **Initial Inpatient Care** |  |  |  |  |
| Length of Stay^b^ | 23.4 | 1.3 | 0.1 | <0.001 |
| Discharge Status | 24.8 |  |  |  |
| Home |  | Reference | - | - |
| Facility^c^ |  | 64.0 | 3.3 | <0.001 |
| Other / Unknown |  | 28.1 | 2.3 | <0.001 |
| Diagnostic Procedure |  |  |  |  |
| CT Scan | 38.4 | -12.9 | 1.4 | <0.001 |
| MRI | 25.6 | 17.6 | 1.6 | <0.001 |
| X-Ray | 25.4 | 5.9 | 1.2 | <0.001 |
| Surgical Procedure | 25.0 | 16.5 | 1.4 | <0.001 |
| Abbreviations: TBI, Traumatic Brain Injury; CT Scan, Computerized Tomography Scan; MRI, Magnetic Resonance Imaging.  SE, Standard Error   1. Age at initial TBI diagnosis is reported in years. 2. Length of stay is reported in days. 3. Facility includes: long-term care, psychiatric, rehab, and skilled nursing facilities. | | | | |

**Supplementary Table 6.** Simple linear regression models to predict total future claims within five years based on initial inpatient treatment.

|  | **5-years**  (n = 1,775) | | | |
| --- | --- | --- | --- | --- |
| **Model** | **Intercept** | **β** | **SE** | **p-value** |
| **Demographics** |  |  |  |  |
| Age^a^ | 61.8 | 0.6 | 0.4 | 0.13 |
| Sex | 67.0 |  |  |  |
| Male |  | Reference | - | - |
| Female |  | 2.4 | 5.1 | 0.64 |
| Geographic Region | 72.7 |  |  |  |
| Northeast |  | -8.2 | 8.2 | 0.31 |
| Central |  | -6.3 | 6.2 | 0.30 |
| South |  | Reference | - | - |
| West |  | -10.9 | 6.4 | 0.09 |
| TBI Severity | 70.6 |  |  |  |
| Mild |  | Reference | - | - |
| Moderate |  | -7.0 | 5.3 | 0.19 |
| Severe |  | -2.0 | 7.5 | 0.79 |
|  |  |  |  |  |
| **Initial Inpatient Care** |  |  |  |  |
| Length of Stay^b^ | 55.3 | 3.0 | 0.2 | <0.001 |
| Discharge Status | 62.1 |  |  |  |
| Home |  | Reference | - | - |
| Facility^c^ |  | 90.9 | 13.6 | <0.001 |
| Other / Unknown |  | 43.4 | 9.7 | <0.001 |
| Diagnostic Procedure |  |  |  |  |
| CT Scan | 85.0 | -23.0 | 5.6 | <0.001 |
| MRI | 61.6 | 37.6 | 6.5 | <0.001 |
| X-Ray | 60.1 | 14.4 | 4.8 | 0.003 |
| Surgical Procedure | 61.4 | 31.2 | 6.0 | <0.001 |
| Abbreviations: TBI, Traumatic Brain Injury; CT Scan, Computerized Tomography Scan; MRI, Magnetic Resonance Imaging.  SE, Standard Error   1. Age at initial TBI diagnosis is reported in years. 2. Length of stay is reported in days. 3. Facility includes: long-term care, psychiatric, rehab, and skilled nursing facilities. | | | | |

**Supplementary Table 7.** Simple linear regression models to predict total future claims within ten years based on initial inpatient treatment.

|  | **10-years**  (n = 211) | | | |
| --- | --- | --- | --- | --- |
| **Model** | **Intercept** | **β** | **SE** | **p-value** |
| **Demographics** |  |  |  |  |
| Age^a^ | 104.8 | 0.6 | 2.4 | 0.81 |
| Sex | 86.0 |  |  |  |
| Male |  | Reference | - | - |
| Female |  | 62.9 | 27.4 | 0.02 |
| Geographic Region | 92.2 |  |  |  |
| Northeast |  | -18.6 | 43.60 | 0.67 |
| Central |  | 28.5 | 35.1 | 0.42 |
| South |  | Reference | - | - |
| West |  | 60.6 | 34.6 | 0.08 |
| TBI Severity | 104.1 |  |  |  |
| Mild |  | Reference | - | - |
| Moderate |  | 16.3 | 29.3 | 0.58 |
| Severe |  | -3.2 | 40.4 | 0.94 |
|  |  |  |  |  |
| **Initial Inpatient Care** |  |  |  |  |
| Length of Stay^b^ | 78.3 | 7.6 | 1.2 | <0.001 |
| Discharge Status | 101.3 |  |  |  |
| Home |  | Reference | - | - |
| Facility^c^ |  | 126.2 | 97.5 | 0.20 |
| Other / Unknown |  | 82.8 | 51.8 | 0.11 |
| Diagnostic Procedure |  |  |  |  |
| CT Scan | 122.5 | -19.6 | 28.2 | 0.49 |
| MRI | 108.4 | 9.2 | 40.1 | 0.82 |
| X-Ray | 94.2 | 31.1 | 26.8 | 0.25 |
| Surgical Procedure | 88.9 | 101.0 | 32.5 | 0.002 |
| Abbreviations: TBI, Traumatic Brain Injury; CT Scan, Computerized Tomography Scan; MRI, Magnetic Resonance Imaging.  SE, Standard Error   1. Age at initial TBI diagnosis is reported in years. 2. Length of stay is reported in days. 3. Facility includes: long-term care, psychiatric, rehab, and skilled nursing facilities. | | | | |

**Supplementary Table 8.** Simple linear regression models to predict total future cost^a^ within one year based on initial inpatient treatment.

|  | **1-year**  (n = 5,011) | | | |
| --- | --- | --- | --- | --- |
| **Model** | **Intercept** | **β** | **SE** | **p-value** |
| **Demographics** |  |  |  |  |
| Age^b^ | $16,561 | $1,105 | $237 | < 0.001 |
| Sex | $27,414 |  |  |  |
| Male |  | Reference | - | - |
| Female |  | -$628 | $3,070 | 0.84 |
| Geographic Region | $30,547 |  |  |  |
| Northeast |  | -$10,181 | $4,990 | 0.04 |
| Central |  | -$2,874 | $3,689 | 0.44 |
| South |  | Reference | - | - |
| West |  | -$6,624 | $3,780 | 0.08 |
| TBI Severity | $23,833 |  |  |  |
| Mild |  | Reference | - | - |
| Moderate |  | $5,322 | $3,186 | 0.10 |
| Severe |  | $10,404 | $4,416 | 0.02 |
|  |  |  |  |  |
| **Initial Inpatient Care** |  |  |  |  |
| Length of Stay^c^ | $17,640 | $2,279 | $131 | <0.001 |
| Discharge Status | $19,258 |  |  |  |
| Home |  | Reference | - | - |
| Facility^d^ |  | $138,653 | $8,087 | <0.001 |
| Other / Unknown |  | $55,199 | $5,678 | <0.001 |
| Diagnostic Procedure |  |  |  |  |
| CT Scan | $35,673 | -$11,197 | $3,389 | <0.001 |
| MRI | $21,737 | $31,251 | $3,807 | <0.001 |
| X-Ray | $20,586 | $11,901 | $2,924 | <0.001 |
| Surgical Procedure | $20,655 | $29,211 | $3,466 | <0.001 |
| Abbreviations: TBI, Traumatic Brain Injury; CT Scan, Computerized Tomography Scan; MRI, Magnetic Resonance Imaging.  SE, Standard Error   1. Costs were reported in dollars (standardized to the fiscal year 2018) and rounded to the nearest whole dollar. 2. Age at initial TBI diagnosis is reported in years. 3. Length of stay is reported in days. 4. Facility includes: long-term care, psychiatric, rehab, and skilled nursing facilities. | | | | |

**Supplementary Table 9.** Simple linear regression models to predict total future cost^a^ within five years based on initial inpatient treatment.

|  | **5-years**  (n = 1,775) | | | |
| --- | --- | --- | --- | --- |
| **Model** | **Intercept** | **β** | **SE** | **p-value** |
| **Demographics** |  |  |  |  |
| Age^b^ | $25,674 | $1,931 | $520 | < 0.001 |
| Sex | $46,308 |  |  |  |
| Male |  | Reference | - | - |
| Female |  | -$6,279 | $6,474 | 0.33 |
| Geographic Region | $54,556 |  |  |  |
| Northeast |  | -$23,373 | $10,365 | 0.02 |
| Central |  | -$17,449 | $7,874 | 0.03 |
| South |  | Reference | - | - |
| West |  | -$16,324 | $8,132 | 0.05 |
| TBI Severity | $40,150 |  |  |  |
| Mild |  | Reference | - | - |
| Moderate |  | $3,497 | $6,744 | 0.60 |
| Severe |  | $19,353 | $9,479 | 0.04 |
|  |  |  |  |  |
| **Initial Inpatient Care** |  |  |  |  |
| Length of Stay^c^ | $30,383 | $3,305 | $306 | <0.001 |
| Discharge Status | $35,462 |  |  |  |
| Home |  | Reference | - | - |
| Facility^d^ |  | $169,001 | $17,068 | <0.001 |
| Other / Unknown |  | $48,708 | $12,225 | <0.001 |
| Diagnostic Procedure |  |  |  |  |
| CT Scan | $51,848 | -$10,423 | $7,124 | 0.14 |
| MRI | $35,684 | $50,830 | $8,268 | <0.001 |
| X-Ray | $31,448 | $23,766 | $6,189 | <0.001 |
| Surgical Procedure | $34,855 | $44,684 | $7,592 | <0.001 |
| Abbreviations: TBI, Traumatic Brain Injury; CT Scan, Computerized Tomography Scan; MRI, Magnetic Resonance Imaging.  SE, Standard Error   1. Costs were reported in dollars (standardized to the fiscal year 2018) and rounded to the nearest whole dollar. 2. Age at initial TBI diagnosis is reported in years. 3. Length of stay is reported in days. 4. Facility includes: long-term care, psychiatric, rehab, and skilled nursing facilities. | | | | |

**Supplementary Table 10.** Simple linear regression models to predict total future cost^a^ within ten years based on initial inpatient treatment.

|  | **10-years**  (n = 211) | | | |
| --- | --- | --- | --- | --- |
| **Model** | **Intercept** | **β** | **SE** | **p-value** |
| **Demographics** |  |  |  |  |
| Age^b^ | $39,787 | $1,328 | $1,407 | 0.35 |
| Sex | $71,123 |  |  |  |
| Male |  | Reference | - | - |
| Female |  | $32,169 | $16,400 | 0.05 |
| Geographic Region | $44,528 |  |  |  |
| Northeast |  | -$24,389 | $25,963 | 0.35 |
| Central |  | $5,290 | $20,928 | 0.80 |
| South |  | Reference | - | - |
| West |  | $37,075 | $20,626 | 0.07 |
| TBI Severity | $47,416 |  |  |  |
| Mild |  | Reference | - | - |
| Moderate |  | $5,755 | $17,536 | 0.74 |
| Severe |  | $10,706 | $24,149 | 0.66 |
|  |  |  |  |  |
| **Initial Inpatient Care** |  |  |  |  |
| Length of Stay^c^ | $31,413 | $4,730 | $697 | <0.001 |
| Discharge Status | $42,509 |  |  |  |
| Home |  | Reference | - | - |
| Facility^d^ |  | $213,824 | $56,419 | <0.001 |
| Other / Unknown |  | $62,631 | $29,945 | 0.04 |
| Diagnostic Procedure |  |  |  |  |
| CT Scan | $59,499 | -$12,849 | $16,868 | 0.45 |
| MRI | $50,085 | $7,545 | $23,947 | 0.75 |
| X-Ray | $38,347 | $25,661 | $15,937 | 0.11 |
| Surgical Procedure | $34,852 | $79,135 | $19,096 | <0.001 |
| Abbreviations: TBI, Traumatic Brain Injury; CT Scan, Computerized Tomography Scan; MRI, Magnetic Resonance Imaging.  SE, Standard Error   1. Costs were reported in dollars (standardized to the fiscal year 2018) and rounded to the nearest whole dollar. 2. Age at initial TBI diagnosis is reported in years. 3. Length of stay is reported in days. 4. Facility includes: long-term care, psychiatric, rehab, and skilled nursing facilities. | | | | |

**Supplementary Table 11**. Linear mixed model parameters for future medical claims and treatment costs following pediatric TBI by TBI diagnosis at initial admission.

| **Model** | **Parameter**  **Name** | **β** | **Lower 95% Confidence Interval** | **Upper 95% Confidence Interval** | **Random Effects Standard Deviation** | **Fixed Effects Standard Deviation** |
| --- | --- | --- | --- | --- | --- | --- |
| **Future Claims** |  |  |  |  |  |  |
| Total | Intercept (Patient) | 21.1 | 19.0 | 23.1 | 65.0 | 1.0 |
|  | Year | 9.4 | 9.1 | 9.7 | - | 0.1 |
|  | Mild | Reference | - | - | - | - |
|  | Moderate | -6.6 | -10.8 | -2.4 | - | 2.1 |
|  | Severe | 0.2 | -5.6 | 6.1 | - | 3.0 |
| TBI^a^ | Intercept (Patient) | 6.3 | 5.1 | 7.4 | 22.2 | 0.6 |
|  | Year | 0.7 | 0.6 | 0.9 | - | 0.1 |
|  | Mild | Reference | - | - | - | - |
|  | Moderate | 0.8 | -0.8 | 2.5 | - | 0.8 |
|  | Severe | 2.1 | -0.2 | 4.4 | - | 1.2 |
| Non-TBI^b^ | Intercept (Patient) | 18.7 | 16.4 | 20.9 | 52.2 | 1.2 |
|  | Year | 8.8 | 8.6 | 9.1 | - | 0.1 |
|  | Mild | Reference | - | - | - | - |
|  | Moderate | -8.2 | -11.6 | -4.7 | - | 1.7 |
|  | Severe | -2.6 | -7.3 | 2.2 | - | 2.4 |
| **Future Costs**^c^ |  |  |  |  |  |  |
| Total | Intercept (Patient) | $23,712 | $16,765 | $30,267 | $168,337 | $2,583 |
|  | Year | $4,148 | $3,574 | $4,722 | - | $293 |
|  | Mild | Reference | - | - | - | - |
|  | Moderate | $302 | -$10,329 | $10,933 | - | $5,425 |
|  | Severe | $10,079 | -$4,683 | $24,840 | - | $7,532 |
| TBI^a^ | Intercept (Patient) | $10,377 | $7,781 | $12,973 | $58,872 | $1,325 |
|  | Year | $566 | $478 | $653 | - | $45 |
|  | Mild | Reference | - | - | - | - |
|  | Moderate | $4,476 | $505 | $8,346 | - | $2,001 |
|  | Severe | $7,239 | $1,889 | $12,589 | - | $2,730 |
| Non-TBI^b^ | Intercept (Patient) | $15,496 | $9,575 | $22,000 | $155,720 | $3,319 |
|  | Year | $3,764 | $3,172 | $4,355 | - | $302 |
|  | Mild | Reference | - | - | - | - |
|  | Moderate | -$4,731 | -$14,637 | $5,176 | - | $5,055 |
|  | Severe | $2,426 | -$11,346 | $16,198 | - | $7,028 |
| Abbreviation: TBI, Traumatic Brain Injury   1. TBI-related measurements were identified as claims with a TBI-related ICD code. 2. Non-TBI related measurements were identified as claims without a TBI-related ICD code. 3. Costs were reported in dollars (standardized to the fiscal year 2018) and rounded to the nearest whole dollar. | | | | | | |

**Supplementary Figure 2**. Linear mixed models of total future medical claims and future treatment cost.

Abbreviation: TBI, Traumatic Brain Injury

1. Models include Total Medical Claims (A) and Total Cost (B). Costs were reported in dollars (standardized to the fiscal year 2018) and rounded to the nearest whole dollar.
